# Environmental Epigenetic Changes, as Risk Factors for the Development of Diseases in Children: A Systematic Review

**DOI:** 10.29024/aogh.909

**Published:** 2018-07-27

**Authors:** Isabel Alvarado-Cruz, Jorge A. Alegría-Torres, Nereida Montes-Castro, Octavio Jiménez-Garza, Betzabet Quintanilla-Vega

**Affiliations:** 1 Toxicology Department, Cinvestav, Mexico City, MX; 2 Pharmacy Department, University of Guanajuato, Guanajuato, MX; 3 Health Sciences Division, University of Guanajuato, Leon Campus, Leon, Guanajuato, MX

## Abstract

**Background:**

Children are susceptible to environmental contaminants and are at risk of developing diseases, more so if the exposure begins at an early age. Epidemiological studies have postulated the hypothesis of the fetal origin of disease, which is mediated by epigenetic changes. Epigenetic marks are inheritable; they modulate the gene expression and can affect human health due to the presence of environmental factors.

**Objective:**

This review focuses on DNA-methylation and its association with environmental-related diseases in children.

**Methods:**

A search for studies related to DNA-methylation in children by pre- or post-natal environmental exposures was conducted, and those studies with appropriate designs and statistical analyses and evaluations of the exposure were selected.

**Findings:**

Prenatal and early life environmental factors, from diet to exposure to pollutants, have been associated with epigenetic changes, specifically DNA-methylation. Thus, maternal nutrition and smoking and exposure to air particulate matter, polycyclic aromatic hydrocarbons, arsenic, heavy metals, persistent organic pollutants, and some endocrine disrupters during pregnancy have been associated with genomic and gene-specific newborns’ DNA-methylation changes that have shown in some cases sex-specific patterns. In addition, these maternal factors may deregulate the placental DNA-methylation balance and could induce a fetal reprogramming and later-in-life diseases.

**Conclusions:**

Exposure to environmental pollutants during prenatal and early life can trigger epigenetic imbalances and eventually the development of diseases in children. The integration of epigenetic data should be considered in future risk assessments.

## Introduction

Environmental pollutants represent a health hazard of global concern because of their chronic exposure and the susceptibility of some groups, such as children. A critical window of vulnerability is the pregnancy period, because intrauterine life defines the health status of adulthood and many risks for developing diseases in late life could be prevented [1].

Epigenetics is the study of the heritable changes in gene expression that occur without a change in the DNA sequence. Epigenetic modifications, including DNA-methylation, regulate critical cellular functions from gene imprinting to gene reprogramming. The most studied epigenetic mechanism is DNA-methylation, which is the addition of a methyl group to carbon-5 of the cytosine in CpG dinucleotides to form 5-methyl cytosine (5 mC) through the action of DNA-methyl transferases (DNMT) [2]. Methylation of repetitive elements LINE 1 and Alu have been widely used as surrogate markers of global DNA-methylation [3]. Most studies regarding epigenetic changes have been conducted in blood; however, blood cells as source for the DNA-methylation analysis has recently been discussed due to the differences in DNA-methylation levels among tissues and cellular types [4].

Epidemiological studies have postulated the hypothesis of the fetal origin of disease, which is mediated by epigenetic changes related to external factors [1]. The epigenome undergoes extensive reprogramming throughout fetal development at gametogenesis and early embryo preimplantation [5], representing vulnerable stages to environmental exposures.

**Figure 1:**
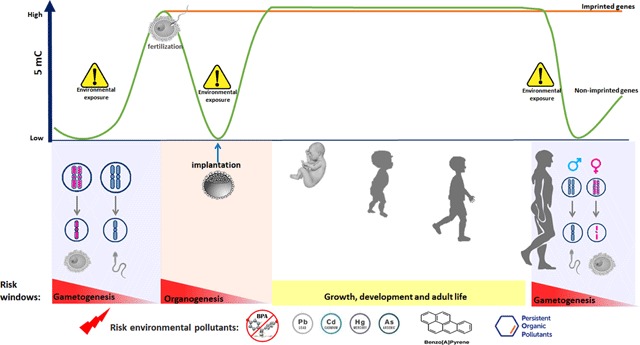
**Susceptibility windows of DNA-methylation due to environmental pollutants.** The epigenome undergoes reprogramming at two relevant stages, the gametogenesis and the early embryo preimplantation representing vulnerable stages to enviornemental exposures. During gametogenesis and fertilization, a general demethylation followed by a re-methylation process occur, with the exception of imprinted genes. DNA-methylation patterns are reestablished by DNA methyl-transferases DNMT3a and DNMT3b [1]. 5 mC, 5-methylcytosine. BPA, bisphenol A. Cd, cadmium. As arsenic. Pb, lead. Hg, mercury.

Some studies in newborns and children have shown the association between DNA-methylation changes and the exposure to environmental factors. However, the interpretation must be taken with caution, because the changes could be reverted; therefore, there is a demand for cohort studies that evaluate the DNA-methylation changes throughout an individual’s life. Variables, such as age, gender, race, ethnicity, folate intake, cellular type, and different environmental exposures must be considered to find discover strong associations [6,7]. Figure 1 shows the susceptibility windows of DNA-methylation to pollutants.

A literature search was done through PubMed, and those papers concerning pregnant women or children reporting changes in global or specific DNA-methylation associated with maternal influences including diet, smoking, and the exposure *in utero* or early in life to pollutants, such as air particulate matter (PM), metals, polycyclic aromatic hydrocarbons (PAHs), persistent organic pollutants (POPs), and some endocrine disrupters, were analyzed. Papers that followed adequate designs and statistical analyses with exposure data were eligible. The review is organized by type of pollutant, the influence on the DNA-methylation patterns (global or specific), and the susceptibility to develop diseases.

## Diet as a Risk Factor for Epigenetic Modifications

Altered maternal nutrition and environmental factors during pre- and early post-natal development can trigger persistent changes that create a predisposition to the development of diseases in adulthood [8,9,10,11]. The major sources of methyl groups from food are methionine, folate, and choline, and the metabolic pathways of these molecules intersect at the formation of methionine from homocysteine. The impairment of the metabolism of these intermediates result in a disturbed availability of the methyl group from S-adenosylmethionine and, subsequently, in incorrect DNA-methylation [12].

Dietary folate is the most studied micronutrient in terms of global and gene-specific methylation changes. Genes such as the insulin-like growth factor 2 (*IGF2*, a maternal imprinted gene) are modified by folic acid intake before and during pregnancy, which is associated with lower methylation at DNA sequences regulating *IGF2* expression [13]. Choline and betaine have demonstrated an influence on DNA-methylation in brain tissue in several *in vivo* models, because choline deficiency is associated with altered neurogenesis followed by decreased memory function [14]. Whereas, *IGF2* methylation was inversely related to maternal serum B12 levels [15]. Maternal nutrition is one of the environmental factors most studied with regard to epigenetic changes, and information about it is well compiled in some reports [16,17]. Therefore, this review will focus on toxic environmental pollutants as modifiers of the DNA-methylation status.

## Effects of Maternal Smoking on Epigenetic Changes in the Offspring

Among the long-term risks associated with parental smoking is the development of cancer in the offspring through epigenetic mechanisms [18,19,20]. The most common epigenetic effect associated with prenatal tobacco smoke exposure is DNA-methylation (Table 1). One of the first reports was a longitudinal study performed in 272 children, where a hypomethylation of AluYb8 was associated with *in utero* tobacco smoke exposure [21]. However, an increase in LINE1 methylation was observed only in individuals carrying the *GSTM1*-positive genotype of the detoxifying enzyme glutathione-S-transferase M-type. This suggests that tobacco smoke could have different epigenetic effects depending on individual genetic diversity [21]. Regarding gene-specific methylations, a hypermethylation on gene promotors involved in the detoxification of tobacco smoke was detected in 272 children from the Children’s Health Study who had been exposed to tobacco *in utero*, specifically on *AXL* (AXL receptor tyrosine kinase), *PTPRO* (protein tyrosine phosphatase, receptor type O), *KLK11* (kallikrein-related peptidase 11), and *TGFB3* (transforming growth factor beta 3) genes [21]. Conversely, a study performed in 90 individuals from a birth cohort of women born in New York City did not find a correlation between prenatal smoke exposure and LINE1 or Alu methylation but found a negative correlation with *SAT*2 (spermidine/spermine N1-acetyltransferase family member 2) [22].

**Table 1:** Methylation changes associated with smoking, POP, phthalates, and BPA exposure during pregnancy and childhood.

Xenobiotic	Population	Sample	Exposure association		Reference

			Change	Sequence	

Prenatal exposure to tobacco smoke	Children from USA (n = 348)	Buccal cells	+	LINE1^a^, *AXL, PTPRO*	[21]
			–	AluYb8	
	Women from USA (n = 90)	Peripheral blood granulocytes	–	*SAT2*	[22]
	Mother-offspring pairs from UK (n = 800)	Cord and blood cells	+	*GFI1^b^, ATP9 A^b^, AHRR, CYP1 A1, CNTNAP2*	[23]
			–	*KLF13^b^, MYO1G*	
	Pregnant women from USA (n = 34)	Placenta	–	*CYP1 A1*	[28]

PBDE47	Mother-newborn pairs (n = 46)	Maternal and cord blood cells	–	*TNFα*	[77]
o,p’-DDTs			–	Alu^c^	
o,p’-DDT, p,p’-DDT p,p’-DDE and PBDEs	Children at birth and/or age 9 (n = 358)	Blood cells	–	LINE1^d^	[83]
			+	LINE1^e^	

Phthalates	Children from Taiwan (n = 256)	Blood cells	+	*TNF-α*	[86]
	Children from USA (n = 235)	Cord blood and blood cells	+/–	LINE1, Alu	[87]
	Children from USA (n = 336)	Cord blood	+	*IRAK4, ESM1, BRCA1, LASP1, IFT140*	[88]
	Mother-newborn pairs from China (n = 119)	Placenta	–	LINE1	[89]
	Mother-newborn pairs from China (n = 220)		–	*IGF2*	[90]
	Mother-newborn pairs from USA (n = 179)		–	*IGF2*	[91]

Bisphenol A	Preadolescent girls from Egypt (n = 60)	Buccal cells	–	X chromosome	[92]
	Mother-newborn pairs from USA (n = 81)	Cord blood	+	*BDNF*	[93]
	Fetuses from USA (n = 12)	Placenta	+/–	LINE1	[94]


+ Increased methylation. – Decreased methylation. ^a^Only in *GSTM1* (Glutathione-S-transferase type M1)-present children. ^b^Methylation changes were reverted over time. ^c^At birth. ^d^Low level of exposure to DDTs plus PBDEs, at birth. ^e^High level of exposure to DDTs plus PBDEs at birth. LINE1 (Long interspersed nuclear element 1). AluYb8 (Alu element members of the Yb8 family). *AXL* (AXL receptor tyrosine kinase). *PTPRO* (Protein tyrosine phosphatase, receptor type O). *SAT*2 (Spermidine/spermine N1-acetyltransferase family member 2). *GFI1* (Growth factor independent 1 transcriptional repressor). *ATP9 A* (ATPase phospholipid transporting 9 A (putative)). *AHRR* (Aryl-hydrocarbon receptor repressor). *CYP1 A1* (Cytochrome P450 family 1 subfamily A member 1). *CNTNAP2* (Contactin Associated Protein-Like 2. *KLF13* (Kruppel like factor 13). *MYO1G* (Myosin 1G). *TNFα* (Tumor necrosis factor alpha). *IRAK4* (interleukin-1 receptor-associated kinase 4); *ESM1* (endothelial cell-specific molecule 1). *BRCA1* (breast cancer 1). *LASP1* (LIM and SH3 Protein 1). *IFT140* (Intraflagellar Transport 140). *IGF2* (Insulin-like growth factor 2). *BDNF* (Brain-derived neurotrophic factor). PBDE (Polybrominated diphenyl ethers). DDT (Dichlorodiphenyltrichloroethane). DDE (Dichlorodiphenyldichloroethylene).

A longitudinal study was conducted in 800 mother-newborn pairs from the ALSPAC Cohort in Southwest England where DNA-methylation were evaluated at birth, 7 years, and 17 years and showed that DNA-methylation changes were reversible for some genes related to development and metabolism, whereas some methylated CpG sites persisted for other genes like *AHRR* (aryl-hydrocarbon receptor repressor), *MYO1G* (myosin IG), *CYP1 A1* (cytochrome P450 family 1 subfamily A member 1), and *CNTNAP2* (contactin associated protein-like 2). Another relevant finding was that the maternal smoking impact was stronger than that of paternal smoking in the post-natal stage [23].

Regarding the epigenetic impact on the placental tissue, nicotine can induce changes in the methylation of more than 7000 fetal genes; these modifications can be preserved for more than three decades after exposure [24,25]. According to Banik et al [26], some of these epigenetically modified placental genes could negatively influence neurodevelopment in the offspring, including the serotonin receptor gene *HTR2 A* [27], *CYP1 A1* [28], the glucocorticoid receptor *NR3C1*, and the corticosteroid dehydrogenase isozyme (*HSD11B2*) [29] genes. Complementarily, a genome-wide scale study performed in 36 placental samples with equal proportions of smokers and nonsmokers showed a correlation between CpG methylation changes and placental transcriptional regulation in 438 genes among smokers, mainly in genes related to oxidative stress [30]. This suggests that deregulation of the placental epigenetic balance could induce a fetal reprogramming.

In a cross-sectional study, cotinine levels were quantified in pregnant women from Valencia, Spain, as a biomarker of smoking, and a remarkable epigenetic effect was found in both maternal and umbilical cord blood, specifically in 31 CpG sites of the exposed group, in the highly relevant adrenomedullin gene, which encodes for a pre-prohormone with important metabolic functions and antimicrobial activities [31].

Avant-garde total epigenome association studies allow for the broadening of the analysis spectrum and the recognition of new genome CpG sites that contribute to a better understanding of the epigenetic mechanisms. Thus, the GECKO Drenthe Birth Cohort performed in cord blood from Dutch children exposed (n = 129) and not exposed (n = 126) to maternal smoking showed a differential methylation profile in *AHRR* (aryl-hydrocarbon receptor repressor), *MYO1G, CYP1 A1, CNTNAP2, LRP5* (LDL receptor related protein 5), and *GFI1* (growth factor independent 1 transcriptional repressor) genes, among others. Particularly, the *GFI1* hypermethylation could explain the effect of prenatal tobacco smoke on birth weight [32]. In fact, there could be a relationship between *in utero* smoking exposure, epigenetic imbalances, and the risk of developing obesity and metabolic syndromes in adulthood [33,34].

## Effects of Fetal and Infant PM Exposure on Global and Gene-specific DNA-methylation

Recently, the IARC has established that air pollution and PM are human carcinogens [35]. PM is a complex mixture of organic and inorganic compounds, such as metals and PAHs. Size and composition determine the particle toxicity [36]. Several studies have shown associations between exposure to PM and DNA-methylation changes, and pregnant women and children are at risk (Table 2).

**Table 2:** Effects of air pollutants on DNA-methylation in newborns and children.

Xenobiotic	Population	Sample	Exposure association		Reference

			Change	Sequence	

PM_2.5_	Mother-newborn pairs from Belgium (n = 240)	Placenta	+	Global methylation	[37]
PM_10_	Fetal growth restriction newborns (n = 80) and normal newborns from China (n = 101)	Placenta	+ –	LINE1 *HSD11B2*	[38]
PM_2.5_	Hispanic and non-Hispanic children from USA (n = 940)	Buccal cells	+	*iNOS, NOS3*	[39,40]
PAHs^a^	Dominican and African American pregnant women-newborn pairs from USA (n = 56)	Cord blood cells	+	*ACSL3*	[41]
PAHs^a^ DNA-PAH adducts	Dominican and African American pregnant women-newborn pairs from USA (n = 164)	Cord blood cells	– +	Global methylation Global methylation	[42]
PAHs^a^	Dominican and African American pregnant women-newborn pairs from USA (n = 53)	Cord blood cells	+	*IFNγ*	[43]
PAHs^b^	Atopic children from USA (n = 256)	Blood cells	+	*FOXP3*	[45]
PAHs^c^ (benzo[b]fluorantene)	Children from Mexico City (n = 150)	Blood cells	+	LINE1	[46]
Pyrene^c^ 8-OHdG			+	*PARP1*	


+ Increased methylation. – Decreased methylation. ETS (Environmental tobacco exposure). LINE1 (Long interspersed nuclear element 1). *HSD11B2* (11-beta-hydroxysteroid dehydrogenase type 2). *iNOS* (Inducible oxide nitric sinthetase). *NOS3* (Nitric oxide synthase 3 expressed in endothelial cells). *ACLS3* (Acyl-CoA synthetase long-chain family member 3). *IL4* (Interleukin 4). *IFNγ* (Interferon gamma). *IFT140* (Intraflagellar transport 140). *FOXP3* (Forkhead box protein P3). *PARP1* (Poly (ADP-ribose) polymerase 1). ^a^PAH concentrations was obtained from personal monitoring. ^b^PAH concentration was obtained from daily at the EPA Supersite. ^c^PAH content in PM_10_.

In a study conducted in 240 placenta samples of women from the ENVIRONAGE Cohort in Flanders, Belgium, DNA-methylation was inversely associated with PM_2.5_ (PM with aerodynamic diameter ≤ 2.5 μm) exposure during the whole pregnancy and during the first trimester; a decrease of 2.13% in global methylation was observed for each 5 μg/m^3^ of PM_2.5_ in the air [37]. With regard to PM_10_ (PM with aerodynamic diameter ≤ 10 μm), the relationship between DNA-methylation and fetal growth restriction was evaluated in 181 placental samples from Wenzhou, China; placenta LINE1 methylation showed a positive association with PM_10_ during the first trimester, and the methylation of *HSD11B2* (hydroxysteroid 11-beta dehydrogenase b2, a gene related to fetal growth) was negatively associated with air PM exposure during the first and second trimesters of pregnancy. These findings were more evident in newborns with fetal growth restriction [38].

On the other hand, Salam et al [39]. reported an association between 7-day average PM_2.5_ exposure and lower methylation of the *NOS* (nitric oxide synthase) promoter in 940 American asthmatic and non-asthmatic children from 6 to 11 years old in New York City; 0.30% lower *NOS* methylation was observed per each 5 mg/m^3^ increase in the 7-day average exposure. Breton et al [40]. evaluated the DNA-methylation in different *NOS* genes (*NOS2 A, NOS3*, and *NOS1*), which are involved in the proinflammatory status and related to PM_2.5_ exposure. Changes in the methylation of specific CpG sites of *NOS2 A* were associated with 7-day, 1-month, 6-month, and 1-year PM_2.5_ concentration averages, and a positive association between 1-year PM_2.5_ exposure and *NOS3* methylation was observed at several CpG sites. The mechanisms linking maternal PM exposure during pregnancy with adverse health outcomes is not clear, and some proposed hypotheses have been related to PM components, such as PAHs and metals, and reactive oxygen species (ROS) produced by air pollutants.

## Polycyclic Aromatic Hydrocarbons (PAHs)

Maternal environmental exposure to PAHs has been demonstrated to have an association with DNA-methylation changes. White cord blood cells from 20 children (10 asthmatic and 10 nonasthmatic) belonging to the CCCEH in New York City were analyzed to identify changes associated with maternal exposure to PAHs through a genome-wide analysis (methylation sensitive restriction fingerprinting) [41]. Thirty sequences were differentially methylated, and six of them were located in CpG islands; the most significant association was observed with the gene expression of acyl-CoA synthetase long-chain family member 3 (*ACSL3*), an enzyme that converts free long-chain fatty-acids into fatty-acyl-CoA esters and thereby plays a key role in lipid biosynthesis and fatty-acid degradation. These data were confirmed in 56 asthmatic children from the same cohort [41]. The relationship between *ACSL3* expression and asthma and the mechanism by which PAHs modify DNA-methylation are not known. In another study performed in the same cohort, PAH exposure was evaluated in 164 women by personal air monitors and the quantification of PAH urinary levels and PAH-DNA adducts [42]. Children with the highest concentration of total PAHs, benzo[a]pyrene, and pyrene concentrations showed the highest DNA-methylation, while the highest PAH-DNA adduct levels were related to an increased global DNA-methylation. In a third report from the CCCEH Cohort, performed in cord blood leukocytes from 53 participants, *IFN*γ (interferon gamma) promoter methylation was associated with maternal PAH exposure [43]. This finding was corroborated *in vitro* in Jurkat and lung adenocarcinoma cell lines exposed to 0.1 and 1 nM of benzo[a]pyrene, in which a promoter hypermethylation and a reduced expression of *IFNγ*, but not interleukin 4 (*IL4*), were observed [43].

A cross-sectional study was performed in the Czech Republic in 400 children, 200 with bronchial asthma and 200 healthy controls from two regions, one polluted (Ostrava, with high levels of benzo[a]pyrene, benzene, NO_2_, PM, and metals) and one control (Prachatice). The results showed that methylation of 9916 CpG sites was significantly different in children from the polluted city, with 58 CpG sites showing lower methylation, suggesting a potentially higher gene expression. Among the altered genes are some involved in the immune response, DNA-protein binding, metabolism of xenobiotics, and signaling pathways. Differences between asthmatic and healthy children by city were observed in *ACSL3* and *ADRB2* (adrenergic beta-2-receptor surface) genes, but this study did not report associations with particular pollutants [44]. In another study performed in Fresno, California, the second-most polluted city in the United States in terms of PM, a group of 256 asthmatic and nonasthmatic children (10–21 years) were included. Exposure to PAHs with 4, 5, or 6 rings was assessed. Increased ambient PAH exposure (mean value of all PAHs) was associated with a hypermethylation and a decreased expression of *FOXP3* (forkhead box P3, a locus involved in atopic or allergic hypersensitivity reactions) and its protein; these changes were associated with the 3-month to 1-year exposures to the PAH mixture with special impact in atopic children. Increments of DNA-methylation in *FOXP3* were associated with IgE plasma levels [45].

Likewise, Alvarado-Cruz et al [46]. reported a positive relationship between PM-related PAH concentrations and LINE1 methylation in a cross-sectional study performed in schoolchildren from the north of Mexico City. This study also analyzed the methylation in CpG sites of some DNA-repair genes and its relationship with the DNA damage. Results showed a higher specific methylation related to some PAH concentrations, particularly the methylation of two CpG sites in the *PARP1* (poly [ADP-ribose] polymerase 1) promoter. Additionally, a 1% increase in the methylation of all CpG sites evaluated in the *PARP1* gene resulted in a 35% increase in DNA oxidative damage (8-OHdG levels) in blood cells. PAH metabolism generates ROS and oxidative damage; however, the exact mechanism by which PAH alter the DNA-methylation is not clear. Experimental evidence showed that oxidative DNA damage and single strand breaks generated by the oxidative stress can increase the *de novo* DNA-methylation [47].

## Epigenetic Alterations by Pre- and Post-natal Exposure to Metals

Several epidemiological studies have shown that the carcinogenic potential of some toxic metals in adult populations may involve epigenetic changes [48,49,50]. However, there are few reports about prenatal or childhood exposures to metals and DNA-methylation (Table 3).

**Table 3:** Effects of metal exposure during pregnancy or childhood on DNA-methylation.

Xenobiotic	Population	Sample	Exposure association		Reference

			Change	Sequence	

Pb	Newborn-mother pairs from Mexico (n = 103)	Cord blood cells	–	Alu, LINE1	[52]
	Children (3 months to 5 years) and mothers from USA (n = 43)	Blood cells	+ –	75 CpG sites 38 CpG sites	[53]
	Children and mothers from USA (n = 35)	Cord blood cells	+/–	564 CpG sites	[54]
Cd	Newborns from Bangladesh (n = 127)	Cord blood cells	+	Global DNA-methylation (newborns: boys)	[59]
			–	Global DNA-methylation (newborns: girls)	
			+	*HISTH4 L, PAX9, APBB3, GAP43*	
			–	*PTPRN2*	
	Mother-newborn pairs from USA (n = 17)	Cord and maternal blood cells	+	Genes related to transcriptional regulation and apoptosis (*PRR13)*	[61]
Hg	Newborns from USA (n = 138)	Cord blood cells	+	85 CpG sites^a^	[63]
	Newborns from USA (n = 321)		–	*PON1*	[65]
As	Newborns from Bangladesh (n = 101)	Cord blood cells	+	Global DNA-methylation	[69]
	Newborns from USA (n = 134)		+	75 CpG sites^a^	[70]
	Newborns from Bangladesh (n = 44)		+	cg00498691	[71]
	Newborns from Bangladesh (n = 127)		–	*LRRC26v, HOXB9, BRSK2*	[72]
	Newborns from Thailand (n = 71)		+	*p53*	[73]
	Newborns from Bangladesh (n = 113)		+	LINE1, *p16*	[74]
V^b^	Children from USA (n = 163)	Buccal cells	–	*IL4, IFNγ*	[76]
	Children from Mexico (n = 150)		+	*APEX, PARP1*	[46]


+ Increased methylation. – Decreased methylation. ^a^Not related to any gene. ^b^V content in PM_10_. *HISTH4 L* (Histone H4 family member). *PAX9* (Paired box 9). *APBB3* (Amyloid beta precursor protein binding family B member 3). *GAP43* (Growth associated protein 43). *PTPRN2* (Protein tyrosine phosphatase, receptor type N2). *PRR13* (Proline-Rich Protein 13). *PON1* (Paraoxonase 1). *LRRC26* (Leucine rich repeat containing 26). *HOXB9* (Homeobox B9). *BRSK2* (BR serine/threonine kinase 2). *p53* (Tumor protein p53). LINE1 (Long interspersed nuclear elements 1). *p16* (Tumor protein p16). *IL4* (Interleukin 4). *IFNγ* (Interferon gamma). *APEX* (Apurinic/apirymidine endonuclease). *PARP1* (Poly (ADP-ribose) polymerase 1).

*Lead* (*Pb*). Lead, an ubiquitous heavy metal, can cross the placenta, representing a risk for the newborn [51]. It is one of the most studied metals in regard to epigenetic modifications. A cross-sectional study was conducted in 103 newborns from the ELEMENT Cohort in Mexico City, in which DNA-methylation was assessed in cord blood and Pb prenatal exposure was obtained by maternal bone Pb levels (mid-tibia shaft and the patella). A negative association between tibia and patella Pb levels and the cord DNA-methylation of Alu and LINE1 was reported [52]. A longitudinal study conducted in 43 children (3 months to 5 years old) and their mothers from a cohort in Michigan, United States, reported their 5 mC profiles [53]. The results showed DNA-methylation changes associated with Pb exposure in both females and males in genes associated with neurogenesis, neuronal differentiation, and ROS-metabolic processes, among others. Different sets of genes presented modifications in males or females, suggesting gender and exposure influences on DNA-methylation.

A second genome-wide methylation study was performed in 35 children and their mothers from the same cohort with PbB above and below 5 µg/dL. Samples from children and mothers at delivery were obtained from the Michigan Neonatal Biobank. The analysis at birth revealed 564 CpG sites that were differentially methylated in children with high PbB compared to low PbB; whereas, the analysis at 3 months to 5 years did not show associations with neonatal or childhood methylation patterns, suggesting that DNA-methylation returns to a normal pattern within the first years of life in low PbB children [54].

An analysis overlapping with the previous results [53] showed a common modification in 6 CpG sites, suggesting that pre- and post-natal Pb exposure can have similar effects on DNA-methylation. These results were corroborated *in vitro* using human embryonic stem cells acutely or chronically treated with Pb (1.5 μM Pb-acetate). Within the 6 CpG sites identified, the brain development-related molecule 1 or N-myc downstream-regulated gene (*NDRG4*) presented an increased methylation. The down-regulation of this gene was associated with reduced levels of the brain-derived neurotrophic factor (BDNF), leading to impaired spatial learning and memory [55]. A hypomethylation of the promoter of the nerve injury-induced protein 2 (*NINJ2*) was observed; this protein is a cell adhesion molecule that acts as a promoter of neurite outgrowth in neurodevelopment [54,56].

Some reports have shown DNA-methylation changes in animal models, which support the hypothesis of the fetal origin of disease. An interesting study conducted in aged primates (23 years old) exposed to Pb during the infantile stage showed an overexpression of the amyloid-protein precursor (APP) involved in Alzheimer’s disease (AD) and high oxidative DNA damage as well as a decrease in DNMT activity. This suggests that epigenetic imprinting in early development influences the expression of AD-related genes and may promote pathogenesis [57].

*Cadmium* (*Cd*). Cadmium is widely distributed in the environment and is considered a carcinogen for humans by the IARC [58]. Few epidemiological studies have evaluated alterations in DNA-methylation by pre- or post-natal Cd exposure. Kippler et al [59]. analyzed the genome-wide DNA-methylation in 127 newborns exposed to Cd from rural Bangladesh. Cd exposure in pregnancy altered the fetal global DNA-methylation in a sex-specific manner. A positive association was observed in boys; whereas, an inverse relationship was observed in girls. Among the 500 CpG sites with stronger correlations between maternal blood Cd (CdB) and cord blood methylation in all newborns, the 5 top genes were histone H4 family member (*HISTH4 L*), paired box 9 (*PAX9*), amyloid beta precursor protein-binding family B member 3 (*APBB3*), growth-associated protein 43 (*GAP43*), and protein tyrosine phosphatase receptor type N2 (*PTPRN2*). Additionally, DNA-methylation of CpG sites in 6 genes was inversely correlated with birth weight. In contrast, a sex-specific analysis showed associations in 40 genes involved in embryonic and organ development in boys and in 105 genes related to cell death. These genes showed a hypermethylation when maternal CdB increased. However, the mechanism that explains the differences by sex can be linked to the X-chromosome silencing [60].

On the other hand, in 17 mother-newborn pairs from a cohort of Durham, North Carolina, Sanders et al [61]. evaluated DNA-methylation and found a subgroup of genes that showed an alteration in fetal DNA-methylation associated with Cd exposure (n = 61) and cotinine (n = 366). Similarly, in maternal DNA, 92 and 134 genes differentially methylated were associated with Cd and cotinine, respectively. Although differentially methylated genes were distinct between mothers and newborns, authors found genes encoding proteins involved in similar functions in fetal and maternal DNA, such as transcriptional regulation and apoptosis, including proline-rich 13 (*PRR13*).

*Mercury* (*Hg*). Mercury has the ability to cross the placenta and is known for its toxicity on neurodevelopment [62]. Recently, Cardenas et al [63]. evaluated the epigenome-wide methylation and its association with Hg exposure using Hg levels in toenails of 138 newborns from New Hampshire, United States. Among the top 100 CpG sites associated with maternal Hg levels, 85% were hypermethylated. In addition, a recent study reported a decrease of 1% to 3.8% in the CpG methylation of paraoxonase 1 gene (*PON1*) in cord blood from males related to maternal blood Hg levels, after stratifying by sex, which persisted during early childhood. PON1 is considered an antioxidant protein involved in metal toxicity; in vitro studies have shown that Hg can inhibit its protective effect [64]. Although no significant changes were observed in DNA-methylation in females, an increase in DNA-methylation of *PON1* was associated with lower cognitive test scores in both sexes. These results suggest that maternal Hg exposure can influence the epigenome of newborns and cause neurodevelopment alterations in childhood [65].

*Arsenic* (*As*). Arsenic is a natural metalloid that contaminates air and water in many countries [66] and is considered a human carcinogen by the IARC [67]. Several studies have shown an association between As exposure and the susceptibility to cancer development in adults throughout epigenetic mechanisms [68], while only few studies are available in children. A study performed in cord blood from a population in Matlab, Bangladesh, (n = 101) showed a positive association between global DNA-methylation and As maternal urine levels [69]. Likewise, Koestler et al [70] showed a hypermethylation in 75% of the top 100 CpG loci associated with As exposure *in utero* in 134 newborns of the New Hampshire Birth Cohort Study. In the same way, another study conducted in 44 newborns of a cohort from Bangladesh, selected according to a range of 15–10 µg/L As in their mother’s drinking water, showed altered DNA-methylation across the epigenome; however, only one CpG site (cg00498691) showed a significant positive association with prenatal As exposure [71].

Another study conducted in a population of 127 newborns from Matlab, Bangladesh, reported that among the 500 CpG sites with the strongest association with urinary As levels in early pregnancy, 277 (55%) CpG sites showed a decreased DNA-methylation, whereas 223 (45%) sites showed an increase. The authors suggest that early exposure may interfere with *de novo* DNA-methylation. Also, these authors showed that As prenatal exposure influenced changes in sex-specific DNA-methylation. For instance, a hypomethylation was observed in CpG sites associated with cancer-related genes: leucine-rich repeat-containing 26 (*LRRC26*), homeobox B9 (*HOXB9*), and BR serine/threonine kinase 2 (*BRSK2*) only in boys, which correlated with an increase in As levels [72]. More studies are needed to evaluate DNA-methylation changes due to As exposure and risk of cancer.

On the other hand, few studies have evaluated alterations in gene-specific DNA-methylation. A study performed in 71 newborns in contaminated areas of Ron Pibul, Thailand, revealed that a high level of As in nails was correlated with DNA-methylation of the *p53* promoter; while no changes were observed in LINE1 methylation [73]. Alterations in tumor suppressor genes can represent an important mechanism for the development of cancer in adult stages.

In contrast, Kile et al [74]. performed a study in 113 newborns from a prospective birth cohort recruited in Sirajdikhan Upazila, Bangladesh, and showed a 1.3% increase in umbilical cord LINE1 methylation in the group with the highest As maternal urine levels and a hypermethylation in the *p16* promoter; however, no differences were found in the methylation of the *p53* promoter.

*Vanadium (V)*. Vanadium is a toxic element that has been associated with high mortality [75]. Some studies have shown the potential effect of V exposure on DNA-methylation. In a cohort of 727 African American and Dominican children diagnosed with asthma, Jung et al [76] demonstrated that V exposure decreases the DNA-methylation of *IL4* and *IFNγ* promoters, and when stratified by overweight status and asthma, a relationship was observed between V exposure and hypomethylation of *NOS2 A*. These effects can lead to gene low expression, which could alter the allergic immune response.

Finally, in a cross-sectional study conducted in 150 children from the north of Mexico City, a positive relationship between V content in PM_10_ and the methylation at apurinic/apirymidine endonuclease (*APEX*) and *PARP1* CpG sites was found [46]. These genes are involved in DNA-repair, so changes in DNA-methylation patterns may contribute to genetic damage and be a possible mechanism for cancer development.

## Exposure to Persistent Organic Pollutants (POPs) and Epigenetic Marks During Pregnancy and Infancy

POPs include the large families of polychlorinated biphenyls (PCBs), organochloride pesticides, such as DDT (dichlorodiphenyltrichloroethane) and its metabolite DDE (dichlorodiphenyldichloroethylene), as well as polybrominated diphenyl ethers (PBDEs), a family of flame-retardant compounds. The high exposure to PBDE is a consequence of the enormous distribution of these compounds in diverse commercial products, and the exposure is greater in the infant population than in the adult population of the United States [77].

Epigenetic alterations due to POP exposure have been primarily studied in adults. For example, a cross-sectional study that included 253 men and 191 women found significant correlations between some POPs in plasma and Alu and LINE1 methylation, depending on the sex. In the case of men, p,p’-DDE, cis-Heptachlor epoxide, and several PCBs negatively correlated with Alu, while p,p’-DDE, PCB153, and PCB180 had positive correlations with LINE1 methylation. On the other hand, for women, only PCB153 and PCB180 showed a negative association with Alu, but p,p’-DDE and almost all PCBs were positively associated with LINE1 methylation. In general, POPs could have a DNA hypomethylating effect on Alu in men, while an increase in LINE1 methylation could be occurring in women, taking into consideration age, body mass index, and smoking and alcohol drinking habits [78].

The relevance of POP exposure during adulthood lies in the impact on germinal cells and the consequent epigenetic footprint in the offspring. Although there is no clear evidence of this, a preliminary study perfomed by Consales et al [79]. in nonoccupationally exposed fertile men from Greenland, Warsaw (Poland), and Kharkiv (Ukraine) found a subtle association between blood levels of PCB-153 and p,p’-DDE and a decreased global methylation in sperm.

Few reports have been published where epigenetic effects are associated with POP exposure during pregnancy or childhood. Because POPs can cross the placenta and reach the newborn through breast milk, exposure in the early stages of life becomes crucial [80,81]. Even neurodevelopmental damage could be influenced by epigenetic mechanisms in scenarios of POP exposure [82]. One study was carried out in 46 mother-newborn binomials from the Boston Birth Cohort where measured blood maternal PBDE concentrations found an effect on tumor necrosis factor-alpha (*TNFα*) expression in cord blood through its promoter methylation. Therefore, these compounds could induce a proinflammatory process through epigenetic changes [77].

On the other hand, another study performed in the birth cohort CHAMACOS from Mexico evaluated the association between global methylation and exposure to POPs in newborns and at nine years of age. The main observation was that Alu methylation was associated with prenatal DDT exposure, while LINE1 methylation changes were observed with the co-exposure to DDTs/PBDEs. POPs showed a hypomethylating effect on LINE1 and Alu during prenatal exposure, and that effect increased at nine years of age, depending on the sex [83]. Both outstanding reports are summarized in Table 4.

## Effects of Some Endocrine Disrupters on DNA-methylation in Children

Phthalates and phenolic substances, such as bisphenols, are widely used in the plastics industry [84,85]. However, they are classified as potential endocrine disrupters, and as such, they have been associated with epigenetic changes (Table 1).

*Phthalates*. In a study performed in 256 Taiwanese children from the CEAS, the methylation of the *TNFα* promoter was lower in children with higher urine 5OH-MEHP (mono (2-ethylhexyl)phthalate values, main metabolite of di-(2-ethylhexyl)phthalate-DEHP); the *TNFα* hypomethylation was inversely correlated with TNFα protein levels. The authors also observed that *TNFα* hypomethylation was associated with asthmatic children; methylation of the CpG site cg10717214 was negatively associated with asthma in children with AA or AG genotypes for the *TNFα* rs1800610, suggesting a genetic susceptibility [86].

Prenatal exposure to phthalates has been studied in several cohorts: in children from the CHAMACOS Cohort (n = 235), maternal urine phthalate metabolites were determined at pregnancy weeks 13 and 26, and an inverse association between prenatal concentrations of MEHP with cord blood methylation of Alu repeats at early and late pregnancy was observed; a similar but weaker association with LINE1 methylation was observed [87]. In 336 mother-newborn pairs from the same cohort, five individual CpG sites hypermethylation was associated with maternal phthalate metabolite concentrations in genes involved in inflammatory response, cancer, endocrine function, and male fertility [88].

Two studies in Chinese cohorts have been reported. In 119 mother-newborn pairs (55 FGR-cases and 64 normal controls). Placental LINE1 methylation was higher in FGR-cases, and it was negatively associated with urinary MEHP and the sum of phthalate metabolites [89]. In another sample of mother-newborn pairs (110 fetal growth restriction-FGR-cases and 110 healthy controls) in Wenzhou, China, the authors found that urinary concentrations of MEHP and mono (2-ethyl-5-oxohexyl) phthalate (MEOHP) were inversely associated with placental *IGF2* promoter methylation. This effect was more evident in FGR newborns [90].

In 176 samples from mother-child pairs from two cohorts in Boston, Massachusetts, the authors evaluated some urinary phthalate metabolites in the first pregnancy trimester and the placental methylation in CpG sites of *IGF2* and *H19* genes (both genes play major roles in embryonic and placental growth). Inverse significant associations were found between some metabolites, and the sum of phthalate metabolites and the methylation of both genes and the *IGF2* hypomethylation showed gender differences with some urine metabolites [91].

Bisphenol A (BPA): Regarding BPA exposure and DNA-methylation, urinary concentrations of BPA and genome-wide methylation were evaluated in saliva samples from 60 Egyptian preadolescent girls (10 to 13 years old). DNA-methylation varied widely among the girls, and higher urinary BPA concentrations were associated with decreased methylation in genes involved in the immune function, transport activity, metabolism, and caspase activity. In particular, hypomethylation of X chromosome was associated with higher urinary BPA, suggesting that BPA exposure may affect human health through epigenetic modification of relevant genes [92].

In a cohort of prenatal exposure to BPA (measured in maternal urine at the thirty-fourth week of pregnancy, n = 81), results showed that exposure to high BPA levels was associated with altered DNA-methylation of the *BDNF* (as a marker of brain development) promoter in cord blood in a sex-specific way (only males showed a significant increased methylation) [93]. Finally, twelve samples from the kidney, liver, and placenta were obtained from fetuses (voluntary pregnancy termination, second trimester), and BPA (free and total) concentrations were determined. Placental free BPA concentration showed a positive correlation with LINE1 methylation, while total BPA concentration negatively correlated with placental LINE1 methylation. No significant correlations were observed in the liver or kidney [94].

## Final Remarks

We conclude that there is a susceptibility to DNA-methylation by the environment during intrauterine development and early life, representing a mechanism through which long-term health effects can be initiated. Maternal influences, including nutrition, smoking, and exposure to environmental pollutants during pregnancy can result in altered DNA-methylation profiles of gene regulatory regions in newborns, many of which translate in risks of developing diseases. Therefore, the methylome is highly sensitive to maternal factors during early development but with long-term impacts. The epigenetic changes can be considered as biomarkers of early exposure and effect, however more evidence is needed to clarify the risk of the epigenetic changes associated with maternal factors or with the childhood environment.
